# Assessing cultural competency among Canadian chiropractors: a cross-sectional survey of Canadian Chiropractic Association members

**DOI:** 10.1186/s12998-023-00474-4

**Published:** 2023-01-12

**Authors:** Nora Bakaa, Danielle Southerst, Pierre Côté, Luciana Macedo, Lisa C. Carlesso, Joy MacDermid, Silvano Mior

**Affiliations:** 1 School of Rehabilitation Sciences, McMaster University, Hamilton, Canada; 2 Institute for Disability and Rehabilitation Research, Ontario Tech University, Oshawa, Canada; 3 School of Physical Therapy, Western University, London, Canada; 4 Division of Research and Innovation, Canadian Memorial Chiropractic College, 6100 Leslie Street, Toronto, ON M2H 3J1 Canada

**Keywords:** Chiropractic, Cultural competence, Cultural diversity, Equity, Health occupations, Rehabilitation

## Abstract

**Background:**

There is a paucity of research assessing cultural competency among Canadian chiropractors. Therefore, the aims of this study were to (1) measure cultural competency among Canadian chiropractors, (2) understand chiropractors’ perspectives of challenges and attitudes regarding the delivery of chiropractic services to equity-seeking communities, and (3) assess contextual factors associated with cultural competency.

**Methods:**

We conducted a cross-sectional survey of members of the Canadian Chiropractic Association (CCA) (May–July 2021). The survey instrument consisted of 57 questions related to demographics, cultural competency, perceptions about health disparities, and challenges in delivery of rehabilitation. Cultural competency was measured using the Cultural Awareness and Sensitivity and Cultural Competence Behaviours subscales of the Cultural Competence Assessment Instrument. We conducted a multivariate linear regression to assess factors that may be associated with cultural competency.

**Results:**

A total of 3143 CCA members responded (response rate of 41%). Mean scores for the Cultural Awareness and Sensitivity subscale were 5.8/7 (95% CI 5.7; 5.8) and 4.2/7 (95% CI 4.1; 4.2) for the Cultural Competence Behaviour subscale. Most chiropractors (72–78%) reported observing important cultural health disparities across various care-related outcomes. Cost of services and language were identified as barriers to providing care to equity-seeking communities. Cultural Awareness and Sensitivity scores were weakly associated with gender (men), years of clinical practice, cultural health disparities, the statement “I think some people have an agenda to look for discrimination even where it does not exist (DEI attitudes),” race (Caucasian), and prior DEI training, (R^2^ = 0.15, *p* < 0.0001). Cultural Competence Behaviour scores were weakly associated with race (Caucasian), cultural health disparities, prior DEI training, increased years of clinical experience, and higher Cultural Awareness and Sensitivity scores (R^2^ = 0.19, *p* < 0.0001).

**Conclusion:**

This study provides the first description of cultural competency within the chiropractic profession in Canada. Findings suggest a gap between knowledge and behaviour and uncover several barriers and challenges that may inform the development of profession-specific training in cultural competence.

**Supplementary Information:**

The online version contains supplementary material available at 10.1186/s12998-023-00474-4.

## Background

Cultural competence can be defined as the consolidation of behaviours, attitudes, and policies that ensure appropriate cultural interactions between systems, agencies, and individuals [1]. More specifically, cultural competency refers to the adequate understanding, appreciation, and respect of cultural differences and similarities among diverse groups [1]. The development of cultural competence among healthcare providers is a crucial step towards achieving equitable delivery of health services. Within the Canadian healthcare system, equity-seeking communities (e.g., LGBTQ2+ , Black people, Indigenous people) often report greater levels of pain and disability and experience greater barriers (e.g., language, cost, etc.) to access of health services [2–8]. The provision of culturally competent care may improve the delivery of health services within the chiropractic profession and reduce cultural health disparities. Several commentaries and narrative reviews have emphasized the need to focus on diversity and cultural competence within the chiropractic profession [9–13]; however, no studies have attempted to measure cultural competency of chiropractors practicing in Canada.

A commonly used conceptual framework for cultural competency explores four key constructs that constitute cultural competency, including: (1) cultural diversity experience; (2) cultural awareness; (3) cultural sensitivity; and (4) cultural competence behaviours [14]. Cultural diversity in experience represents a clinician’s unique personal experience and exposure to equity-seeking communities that may influence their cultural competency. Cultural awareness and sensitivity refer to knowledge, attitudes, values, and beliefs regarding cultural diversity. Lastly, culturally competent behaviours refer to the translation of cultural diversity experiences and cultural awareness and sensitivity into clinical practice.

We developed a survey to assess the 4 constructs of cultural competency in Canadian chiropractors. Results pertaining to cultural diversity experience are reported elsewhere [15]. Therefore, the purpose of this study was to (1) describe the remaining pillars of the cultural competency framework (i.e., cultural awareness and sensitivity, cultural competence behaviours) among Canadian chiropractors, (2) understand chiropractors’ perspectives of the challenges and attitudes regarding the delivery of chiropractic services to equity-seeking communities, and (3) assess the contextual factors potentially associated with cultural competency.

## Methods

We used the Strengthening the Reporting of Observational Studies in Epidemiology (STROBE) and the Checklist for Reporting Results of Internet E-Surveys (CHERRIES) to report this study [16, 17]. Ethics approval was obtained from the Canadian Memorial Chiropractic College (Project # 2,102,003), Hamilton Integrated Research Ethics Board (Project # 13,042), and Ontario Tech University (Project # 16,392).

### Study design

Cross-sectional survey.

### Participants & recruitment

We targeted all registered members of the Canadian Chiropractic Association (CCA) between May 12 and June 24, 2021. The CCA represents approximately 85% of all practicing Canadian chiropractors, with 7721 members as of January 2021. We engaged senior leadership at the CCA to develop a member recruitment strategy. We used a modified tailored design method to develop materials and schedule recruitment [18]. In collaboration with the CCA, a survey link was distributed at specific timepoints (May 12, 16, 26, and 30, June 6, 7 and 12, 2021) via CCA’s social media pages (i.e., Facebook, Instagram), e-newsletter (i.e., BackMatters), and direct member emails. The CCA also engaged with individual provincial associations to promote the survey to maximize recruitment. Additionally, to incentivise participation, survey participants were given the opportunity to enter a draw for one of ten one-year paid CCA memberships.

### Survey

The survey was administered using the online survey platform LimeSurvey© [19], which was licensed and stored at McMaster University. The survey questions were informed by recent literature in cultural competency [20, 21], the authors’ expertise, and in consultation with the CCA, the Canadian Physiotherapy Association (CPA), the CPA’s Global Health Division and Indigenous Health Sub-Committee, and a subject expert from the Centre for Hate, Bias, and Extremism at Ontario Tech University. The survey instrument was also translated into French. Both the English and French versions of the survey were pilot tested among a purposeful sample of 16 Canadian chiropractors that were diverse with respect to age, gender, and years in practice. Results of the small pilot study were used to improve clarity and functionality of the survey instrument.

The final survey included 57 items that were intended to assess the four constructs of cultural competency (i.e., cultural diversity experience, cultural awareness, cultural sensitivity, and cultural competence behaviours) (Additional file 1: Appendix A) [14]. For cultural diversity experience (15 items), we assessed demographic information (e.g., age, gender, race, etc.) and practice-related information (e.g., years in clinical practice, practice setting, etc.). The results of cultural diversity experience are reported separately [15].

Cultural awareness, sensitivity, and competence behaviours were assessed using the two subscales of the Cultural Competence Assessment Instrument (CCAI): cultural awareness and sensitivity (11 items) and cultural competence behaviour (14 items) [22, 23]. The CCAI has been administered to other health care providers (e.g., nurses, surgeons) and shown to have high to moderate quality evidence for content and construct validity, high-quality evidence for internal consistency (Cronbach’s alpha 0.86–0.93), and low-quality evidence for test–retest reliability (ICC 0.82–0.87) [22, 24, 25]. The Cultural Awareness and Sensitivity items are scored on a 7-point agreement scale (e.g., strongly agree to strongly disagree), while the Cultural Competence Behaviour items are scored on a 7-point frequency scale (e.g., always to never). A total score is calculated by summing the scores from individual items, then dividing by total number of items in each scale for a maximum score out of 7. Total scores were only calculated for participant surveys having less than 20% missing responses, as incomplete responses may lead to inadequate interpretation of scales [26]. The Cultural Awareness and Sensitivity and Cultural Competence Behaviour subscales do not have a standardized cut-off; however, scores greater than 5 on each scale are considered high [27].

The remaining questions (17 items) assessed topics relating to the presence of cultural health disparities (i.e., pain and clinical health outcomes, satisfaction with care, and overall quality of life) and barriers to the provision of rehabilitation services (i.e., challenges experienced in the delivery of rehabilitation services to equity-seeking communities, attitudes towards/management of DEI within clinical practice), where each question was scored on a 7-point agreement scale. Additional questions asked about participants’ previous education and training in topics related to DEI.

## Analysis

De-identified data was downloaded from Lime Survey© to Microsoft Excel [28] and then uploaded onto Stata/BE 17 [29] for analysis. Descriptive statistics were reported as frequencies (percentage), means (95% confidence interval (CI) or standard deviations (SD)).

Univariate and multivariate regression analyses were conducted to assess factors associated with the Cultural Awareness and Sensitivity and Cultural Competence Behaviour subscales. Independent variables included Cultural Awareness and Sensitivity scores (for the Cultural Competence Behaviour model only), years of clinical practice experience, race, prior DEI training, community practice population (i.e., rural/remote region, town or smaller region city, or major city), and disagreement with the statements “I think some people have an agenda to look for discrimination even where it does not exist” (DEI attitudes) and “I have observed cultural health disparities in overall quality of life” (cultural health disparities). We did not include age as an independent variable because it was highly correlated with years of practice (r = 0.95). These variables were chosen based on previous literature assessing cultural competency in other health care professions [23, 30, 31], as well as the authors’ prior understanding and experience in this topic. According to the Sex and Gender Equity in Research (SAGER) guidelines, data should be reported by sex/gender, and an analysis of sex/gender differences and/or similarities should be described [32]. Given cultural competency is a social construct, we conducted regression analysis disaggregated by gender. Gender was included as binary categories (woman/man), as the sample size for the remaining gender categories were too small for segregate analyses, and thus, not included in a separate model. We used backwards elimination to achieve the final models, where the model began containing all included independent variables with non-significant variables removed if the significance level was greater than or equal to 0.05 [33]. Regression diagnostics were conducted to ensure the requisite assumptions for multiple regression (e.g., collinearity, homogeneity of variance, normality of residuals, variance inflation). To avoid overfitting our model, we ensured at least 10–15 observations for each independent variable within the model. To determine the influence of significant independent variables on the dependent variable, we used non-standardized beta (β) coefficients.

## Results

**Fig. 1:**
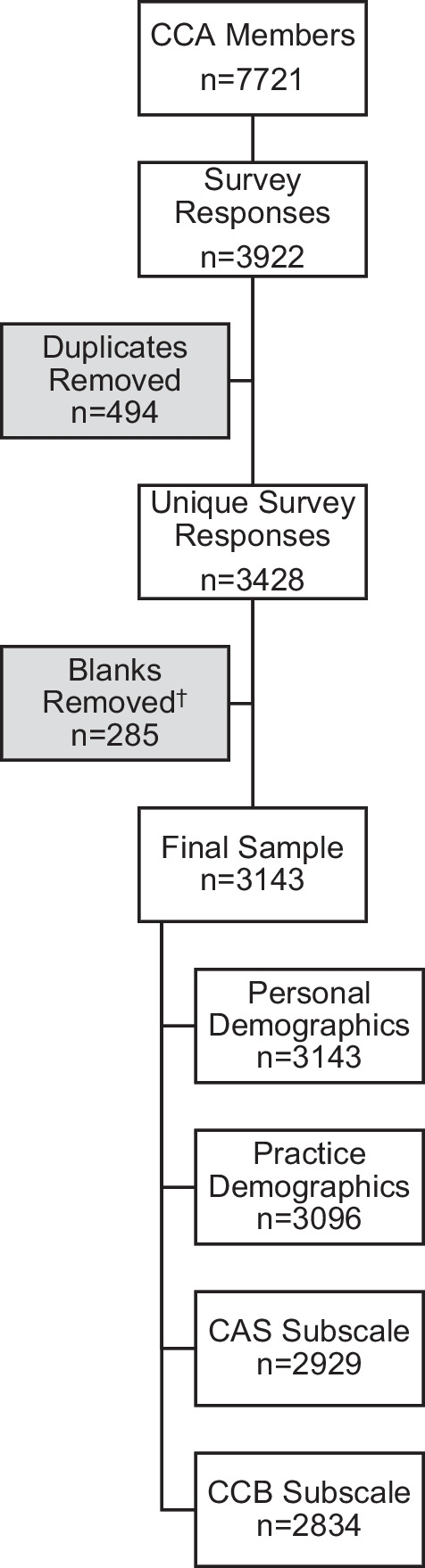
Survey recruitment flow diagram

After the removal of duplicate responses (n = 494), we included 3428 survey responses, accounting for a 41% response rate among CCA members (Fig. 1). Duplicate responses were removed based on a set of a priori rules to account for respondents that may have completed the survey twice (see Additional file 2: Appendix B for more details). Baseline characteristics of the study sample are summarized in Table 1. A more detailed account of demographic factors has been described in a separate manuscript [15].

**Table 1:** Summary demographics of Canadian chiropractors

Demographics	Response category	Survey sample
Age (n = 3100)	Mean (SD); years	44.7 (12.7)
Sex (n = 3100)	Female (%)	45.0
	Male (%)	55.0
Gender (n = 3141)	Woman (%)	45.2
	Man (%)	54.6
	Gender minority (%)^a^	0.2
Indigenous	Yes (%)	0.2
Race (n = 3143)	Black (%)	0.5
	Filipino (%)	0.5
	Latin (%)	0.3
	Southeast Asian (%)	0.6
	Arab (%)	1.1
	Chinese (%)	4.0
	South Asian (%)	5.1
	West Asian (%)	1.0
	Korean (%)	0.8
	Japanese (%)	0.5
	Caucasian (%)	80.3
	Mixed (%)	4.1
	Other (%)	1.2
Years in practice	Mean (SD); years	17.5 (12.3)
Community^b^	Rural/remote (%)	9.7
	Town or smaller regional city (%)	29.3
	Major city (%)	61.0

*SD* Standard deviation

^a^Gender minority categories collapsed due to small sample sizes: (1) trans-man; (2) trans-woman; (3) gender fluid or non-binary; (4) Indigenous or other cultural gender minority (e.g., Two-spirit); (5) identity not listed

^b^Rural/remote = population 1000–10,000; Town or smaller regional city = population 10,000–100,000; Major city (urban/metropolitan/suburban) = population > 100,000

## Cultural competence

The average score for the Cultural Awareness and Sensitivity subscale was 5.8 (95% CI [5.7; 5.8]), whereas the average score on the CCB subscale was 4.2 (95% CI [4.1, 4.2]).

### Cultural awareness and sensitivity subscale

The univariate regression analysis revealed gender (men), race (Caucasian), prior DEI training, years of clinical practice, DEI attitudes, and cultural health disparities were significantly associated with Cultural Awareness and Sensitivity scores (Table 2).

**Table 2:** Univariate analysis for variables associated with cultural awareness and sensitivity

Cultural awareness and sensitivity		
Variable	ß [95% CI]	R^2^
Gender (man)	− 0.25 [− 0.30, − 0.21]	R^2^ = 0.05, F (2, 2856) = 81.13, *p* < 0.0001
Race (Caucasian)	0.10 [0.05, 0.15]	R^2^ = 0.005, F (2, 2857) = 15.34, *p* < 0.0001
Prior DEI training	0.21 [0.21, 0.02]	R^2^ = 0.03, F (2, 2370) = 71.15, *p* < 0.0001
Years of clinical experience	− 0.01 [− 0.01, − 0.01]	R^2^ = 0.03, F (2, 2844) = 90.08, *p* < 0.0001
Community practice population		R^2^ = 0.002, F (2, 2857) = 2.45, *p* > 0.05*
Town or smaller region	0.07 [− 0.00, 0.15]*	
Major city	0.03 [− 0.04, 0.10]*	
Agreement with the statement: *I think some people have an agenda to look for discrimination even where it does not exist*		R^2^ = 0.05, F (2, 2364) = 18.98, *p* < 0.0001
Strongly agree	− 0.19 [− 0.32, − 0.07]	
Agree	− 0.07 [− 0.19, 0.05]*	
Somewhat agree	0.002 [− 0.10, 0.11]*	
Neutral	− 0.03 [− 0.14, 0.08]*	
Somewhat disagree	0.1 [− 0.02, 0.22]*	
Disagree	0.21 [0.09, 0.32]	
Strongly disagree	0.39 [0.25, 0.53]	
*I have observed health disparities in overall quality of life*		R^2^ = 0.03, F (2, 2717) = 40.44, *p* < 0.0001
Never	− 0.31 [− 0.38, − 0.24]	
Unsure	− 0.10 [− 0.16, − 0.03]	

*p > 0.05

When assessing the multivariate regression, there were weak associations between Cultural Awareness and Sensitivity scores and gender, race, prior DEI training, years of clinical practice, DEI attitudes, and cultural health disparities, R^2^ = 0.15, F (14, 2343) = 29.13, *p* < 0.0001 (Table 3). Cultural Awareness and Sensitivity scores were lower (− 0.17) if individuals identified as “man”, were unsure of the presence of cultural health disparities or had never observed them (− 0.09, − 0.28, respectively), had less years of clinical experience (− 0.004), or if individuals strongly agreed with the DEI attitudes statement (− 0.13), keeping all other factors constant. Cultural Awareness and Sensitivity scores were higher if individuals identified as “Caucasian” (0.13), if they had prior DEI training (0.15), or if individuals disagreed (0.14) or strongly disagreed (0.36) with the DEI attitudes statement, keeping all other factors constant. All regression diagnostics were met.

**Table 3:** Multivariate regression coefficients for cultural awareness & sensitivity and cultural competency behaviour scores disaggregated by gender

Independent variables	Total sample	Woman	Man
	ß [95% CI]	ß [95% CI]	ß [95% CI]
*Cultural awareness and sensitivity*			
Model	R^2^ = 0.15	R^2^ = 0.16	R^2^ = 0.05
Gender (n = 2859)			
Man	− 0.17 [− 0.21, − 0.12]	–	–
Race (n = 2859)			
Caucasian	0.13 [0.08, 0.19]	0.08 [0.01,0.16]	0.16 [0.07, 0.23]
Prior DEI training (n = 2372)	0.15 [0.11,0.20]	0.18 [0.12,0.24]	0.12 [0.05, 0.19]
Years of clinical practice (n = 2846)	− 0.004 [− 0.01,0.00]	− 0.003 [− 0.01,0.00]	− 0.01 [− 0.01,0.00]
Agreement with the statement: *I think some people have an agenda to look for discrimination even where it does not exist.* (n = 2372)			
Strongly agree	− 0.13 [− 0.25,0.00]	–	–
Disagree	0.14 [0.03,0.25]	0.18 [0.04,0.32]	–
Strongly disagree	0.36 [0.23,0.49]	0.46 [0.29,0.62]	–
*I have observed health disparities in overall quality of life.* (n = 2720)			
Never	− 0.28[− 0.35, − 0.21]	− 0.34[− 0.44, − 0.24]	− 0.19[− 0.30, − 0.10]
Unsure	− 0.09[− 0.01, 0.00]	− 0.10[− 0.18, − 0.01]	–
*Cultural competence behaviour*			
Model	R^2^ = 0.19	R^2^ = 0.20	R^2^ = 0. 17
Gender (n = 2372)			
Man	− 0.06 [− 0.14,0.02]*	–	–
Race (n = 2518)			
Caucasian	− 0.14 [− 0.24, − 0.04]	–	− 0.14 [− 0.27, − 0.02]
Years of clinical practice (n = 2507)	0.01 [0.00,0.01]	0.01 [0.01,0.02]	–
Prior DEI training (n = 2157)	0.27 [0.18,0.35]	0.31 [0.19,0.43]	0.24 [0.12, 0.35]
Cultural awareness & sensitivity scores (n = 2493)	0.64 [0.57,0.71]	0.66[0.54,0.78]	0.59 [0.50,68]
*I have observed health disparities in overall quality of life.* (n = 2478)			
Never	− 0.37 [− 0.49, − 0.23]	− 0.34 [− 0.54, − 0.13]	− 0.37 [− 0.53, − 0.20]
Unsure	− 0.24 [− 0.38, − 0.11]	− 0.24 [− 0.44, − 0.05]	− 0.28 [− 0.46, − 0.10]

*Gender not statistically significant, but included in the model as a covariate

When the multivariate regression was stratified by gender, prior DEI training, race, years of clinical practice, DEI attitudes and cultural health disparities were associated with Cultural Awareness and Sensitivity scores in women, R^2^ = 0.16, F (12, 1021) = 16.02, *p* < 0.0001. The same variables were significantly associated with Cultural Awareness and Sensitivity scores among men, except for DEI attitudes which was not significant, R^2^ = 0.05, F (5, 1295) = 12.64, *p* < 0.0001.

### Cultural competence behaviour subscale

The univariate regression analysis revealed gender (men), race (Caucasian), prior DEI training, years of clinical practice, DEI attitudes, and cultural health disparities were significantly associated with Cultural Competent Behaviour scores (Table 4).

**Table 4:** Univariate analysis for variables associated with cultural competence behaviour

Cultural competence behaviour		
Variable	ß [95% CI]	R^2^
Gender (man)	− 0.29 [− 0.30, − 0.14]	R^2^ = 0.01, F (2, 2515) = 14.73, *p* < 0.0001
Race (Caucasian)	− 0.11 [− 0.21, − 0.014]	R^2^ = 0.002, F (2, 2516) = 5.04, *p* > 0.05*
Years of clinical experience	− 0.001 [− 0.005, 0.001]*	R^2^ = 0.0003, F (2, 2505) = 0.71, *p* > 0.05*
Prior DEI training	0.42 [0.33, 0.51]	R^2^ = 0.04, F (2, 2155) = 87.43, *p* < 0.0001
Community practice population		
Town or smaller region	0.02 [− 0.13, 0.17]*	R^2^ = 0.002, F (2, 2515) = 2.28, *p* > 0.05*
Major city	0.10 [− 0.04, 0.24]*	
Agreement with the statement: *I think some people have an agenda to look for discrimination even where it does not exist*		
Strongly agree	− 0.25 [− 0.50, − 0.002]	R^2^ = 0.02, F (2, 2149) = 7.81, *p* < 0.0001
Agree	− 0.01 [− 0.25, 0.23]*	
Somewhat agree	0.02 [− 0.19, 0.24]*	
Neutral	0.10 [− 0.12, 0.32]*	
Somewhat disagree	0.11 [− 0.13, 0.36]*	
Disagree	0.32 [0.09, 0.55]	
Strongly disagree	0.42 [0.15, 0.69]	
*I have observed health disparities in overall quality of life*		
Never	− 0.59 [− 0.71, − 0.46]	R^2^ = 0.03, F (2, 2475) = 45.64, *p* < 0.0001
Unsure	-0.27 [− 0.41, − 0.14]	
Cultural awareness and sensitivity scores	0.68 [0.61, − 0.75]	R^2^ = 0.15, F (2, 2491) = 428.87, *p* < 0.0001

*p > 0.05

When assessing the multivariate regression, we found weak associations between race, years of experience, prior DEI training, Cultural Awareness and Sensitivity scores, and cultural health disparities with Cultural Competence Behaviour scores, R^2^ = 0.19, F (8, 2115) = 63.39, *p* < 0.0001 (Table 3). Cultural Competence Behaviour scores were lower (− 0.14) if the individual identified as “Caucasian,” and if they were unsure of the presence of cultural health disparities or had never observed them (− 0.37, − 0.24, respectively), keeping the other factors constant. CCB scores were higher with years of experience (0.01), prior DEI training (0.27), and Cultural Awareness and Sensitivity scores (0.64), respectively, keeping the other factors constant. Gender was not a significant predictor of Cultural Competence Behaviour scores but was controlled for in this model. The only variable that was significant in the univariate analysis, but not significant in the multivariate analysis was gender (men) for cultural competency behaviour subscale. It is likely that gender (men) is only predictive due to its association with other variables within the model. All regression diagnostics were met.

When the multivariate analysis was stratified by gender, years of practice, prior DEI training, Cultural Awareness and Sensitivity scores, and cultural health disparities were associated with Cultural Competence Behaviour scores in women, R^2^ = 0.20, F (5, 909) = 45.13, *p* < 0.0001. Among men, similar variables were associated with Cultural Competence Behaviour scores except for years of practice, which was not significant, and race (Caucasian) which was significant, R^2^ = 0.17, F (5, 1184) = 48.47, *p* < 0.0001.

### Cultural health disparities

Most chiropractors (ranging from 72 to 78%) reported observed health disparities in pain outcomes, clinical outcomes, satisfaction with care, and overall health status due to patients’ cultural differences (Table 5).

**Table 5:** Distribution of observed cultural health disparities in clinical practice

	No (N (%))	Yes (N (%))	Unsure (N (%))
I have observed cultural health disparities in pain outcomes	328 (11.8)	2131 (76.6)	322 (11.6)
I have observed cultural health disparities in clinical outcomes	365 (13.1)	2034 (73.1)	382 (13.7)
I have observed cultural health disparities in satisfaction with care	381 (13.7)	1988 (71.5)	412 (14.8)
I have observed cultural health disparities in overall health status	309 (11.1)	2099 (75.5)	372 (13.4)

### Barriers to rehabilitation

There was a great degree of variability among respondents with some chiropractors agreeing, and others disagreeing with the statements associated with barriers to rehabilitation (Table 6). Barriers to rehabilitation in diverse communities were noted in relation to cost of services, compliance with treatment recommendation, trust in the health care system, and language barriers. Some clinicians also noted that language barriers did not prevent them from providing informed consent/education, and that they did not have difficulty developing treatment plans or building rapport with patients from diverse communities.

**Table 6:** Distribution of responses regarding barriers to providing rehabilitation services to equity seeking groups

	Strongly agree (N (%))	Agree (N (%))	Somewhat agree (N (%))	Neutral (N (%))	Somewhat disagr**e**e (N (%))	Disagree (N (%))	Strongly disagree (N (%))	No opinion (N (%))
The *cost of my services* limits me from providing care for people from some communities and/or cultural groups	319 (11.6)	626 (22.8)	625 (22.7)	286 (10.4)	153 (5.6)	392 (14.2)	240 (8.7)	111 (4.0)
I find it difficult to develop treatment plans that meet the diverse needs of the people in my community	41 (1.5)	164 (6.0)	363 (13.2)	447 (16.2)	365 (13.3)	840 (30.5)	423 (15.4)	109 (4.0)
Patients from some communities and/or cultural groups have difficulty complying with my treatment recommendations (e.g., number of treatment sessions, adherence to exercise, etc.)	135 (5.0)	170 (6.2)	552 (20.1)	796 (29.0)	379 (14.0)	197 (7.2)	369 (13.4)	154 (5.6)
I find it difficult to build rapport with patients who are different from me	5 (0.2)	21 (0.8)	145 (5.3)	195 (7.1)	312 (11.3)	1014 (36.9)	1025 (37.3)	35 (1.3)
Patients from some communities and/or cultural groups have difficulty trusting the health care system	148 (5.4)	501 (18.2)	719 (26.1)	546 (19.9)	172 (6.3)	257 (9.3)	125 (4.5)	283 (10.3)
Language barriers prevent me from providing an informed consent and educating some of my patients to the extent that I would like	79 (2.9)	280 (10.2)	682 (24.8)	314 (11.4)	313 (11.4)	639 (23.2)	356 (12.9)	88 (3.2)
Language barriers impact some of my patients’ ability to comply with care instructions	85 (3.1)	385 (14.0)	835 (30.4)	310 (10.9)	311 (11.3)	504 (18.3)	229 (8.3)	101 (3.7)

### Self-efficacy

There was also significant variability among responses with some chiropractors agreeing and others disagreeing with each statement as it related to self-efficacy and attitudes surrounding diversity, equity, and inclusion (Table 7). Some clinicians were confident in their ability to provide culturally sensitive care to patients, that their actions met current DEI standards, and to approach cultural competency with their staff. Additionally, some clinicians noted that they promote equity and inclusion among staff and colleagues, that they try their best to hire staff that is representative of their community and provide accommodations for patients that speak a different language. While there was a great degree of variability regarding the statement “I think some people have an agenda to look for discrimination even where it does not exist,” approximately 45% agreed to some degree.

**Table 7:** Distribution of responses for self-efficacy and attitudes surrounding DEI in clinical practice

Section 7: self-efficacy regarding DEI	Strongly agree (N (%))	Agree (N (%))	Somewhat agree (N (%))	Neutral (N (%))	Somewhat disagree (N (%))	Disagree (N (%))	Strongly disagree (N (%))	No opinion (N (%))
I am uncertain about how to approach cultural competence with my staff	35 (1.4)	175 (7.2)	385 (15.9)	419 (17.3)	343 (14.2)	600 (24.7)	264 (10.9)	208 (8.6)
I am uncertain about how to provide culturally sensitive care to my patients	22 (0.9)	107 (4.4)	392 (16.1)	330 (13.6)	447 (18.4)	738 (30.4)	309 (12.7)	84 (3.5)
I am uncertain whether my actions or words will be seen as not meeting current standards in diversity, equity, and inclusion	35 (1.4)	172 (7.1)	464 (19.1)	423 (17.4)	384 (15.8)	585 (24.1)	264 (10.9)	102 (4.2)
I think some people have an agenda to look for discrimination even where it does not exist	200 (8.2)	282 (11.6)	609 (25.1)	490 (20.2)	225 (9.3)	339 (14.0)	130 (5.4)	154 (6.3)
I do my best to hire staff that are representative of the community in which I practice	239 (9.8)	680 (28.0)	350 (14.4)	492 (20.3)	40 (1.7)	65 (2.7)	67 (2.8)	496 (20.4)
I accommodate patients who speak a language other than my own (e.g., invite a family member or staff member to translate, translate intake and educational materials, use of translation apps/software, etc.)	1085 (44.7)	909 (37.4)	226 (9.3)	84 (3.5)	26 (1.1)	15 (0.6)	9 (0.4)	75 (3.1)
I promote equity and inclusion among staff and colleagues (e.g., arranging or participating in cultural competency training, etc.)	420 (17.35)	624 (25.7)	266 (11)	537 (22.1)	68 (2.8)	67 (2.8)	55 (2.3)	392 (16.1)

### Training

Most chiropractors (65%) reported not having received any training in DEI. Of those individuals that had received training, most had completed the CCAs DEI training (36%) followed by content covered in university/college courses (16%), continuing education (15%), professional conferences/seminars (13%), separate college/university courses for credit (10%), and employer sponsored programs (9%). Most chiropractors (66%) stated that they were likely to engage in additional training for DEI. When reviewing concepts covered in current training (e.g., health disparities, institutionalized bias, etc.), there was a lack of educational training on these topics, ranging from 18 to 69% (Table 8).

**Table 8:** Distribution of responses for educational concepts that were covered (vs not covered) within entry-level educational programs

	N (%)
Racial and/or ethnic predispositions to genetic-specific illness and disorders	1352 (55.6)
Biological differences between males and females in sex-specific illness and disorders	1671 (68.7)
Psychological and social stresses of living in a discriminatory society	726 (29.9)
Cultural health disparities and utilization of funded health services	807 (33.2)
Cultural health disparities in socioeconomic status, health status and language barriers	981 (40.3)
Cultural health disparities in access to social services, education, employment, and health services	765 (31.5)
Potential for bias in health care delivery to people from diverse communities and/or cultural groups	807 (33.2)
Institutionalized racial and ethnic biases	454 (18.7)
Institutionalized sex and gender roles	506 (20.8)
Intersectionality of the social determinants of health (e.g., race, gender, sexual orientation, ethnicity, etc.)	549 (22.6
None	425 (17.5)

## Discussion

This study provides an initial assessment of cultural competency among Canadian chiropractors as measured by cultural awareness and sensitivity, culturally competent behaviours, and perceived challenges to the delivery of services to equity-seeking groups. In general, chiropractors had higher scores for Cultural Awareness and Sensitivity, and lower scores for Culturally Competent Behaviour. Several variables were weakly associated with each subscale (e.g., gender (men), race (Caucasian), years of clinical experience, cultural health disparities, DEI attitudes, prior DEI training). Additionally, we noted that most chiropractors reported observing important cultural health disparities across various care-related outcomes. Finally, we identified several challenges (e.g., cost of services, development of treatment plans, language barriers) that may influence the delivery of services to equity-seeking groups.

Our findings on cultural competence are similar to those conducted in other professions using the CCAI. Schim et al. (2005) measured cultural competence in hospital-based providers in Ontario and Michigan and found high cultural awareness and sensitivity but lower adoption of culturally competent behaviours [30]. Similar findings were also reported among oncology surgeons in Washington (Mean: CAS = 5.9, CCB = 4.3) [31]. It is unclear whether behaviour measured using a questionnaire translates into behaviour into clinical practice. The results of our study suggest a potential lack of optimal cultural competency training that is practical and applicable to chiropractors. In our study, cultural knowledge in the form of cultural awareness and sensitivity was positively associated with previous training in DEI and culturally competent behaviours. Most chiropractors reported not having received training in DEI, and many were unfamiliar with key concepts of culturally competent care delivery (assumed by response to knowledge of key concepts of cultural competency in current training). These findings are consistent with previous literature assessing cultural competency among other health care providers (e.g., surgeons, nurses) [23, 31]. This may be a significant opportunity for chiropractic organizations (e.g., colleges, regulators, associations) to impact the cultural competency of the chiropractic workforce.

Our study found several factors that were associated with cultural competency. Race and years of health care experience were associated with cultural competency, which differs from previous literature [30]. It is possible that with increased years of experience there is increased exposure to diverse groups, which in turn relates to increased cultural awareness and sensitivity, and cultural competence behaviour. It was interesting to note race (Caucasian) was positively associated with cultural awareness and sensitivity, and negatively associated with cultural competence behaviour. This may suggest a lack of translation of cultural knowledge to behaviour among Caucasians. Consistent with previous literature, gender was associated with cultural awareness and sensitivity [30], and prior DEI training was associated with both provider knowledge and behaviour [23, 31]. Although we did not find differences in DEI training between gender, it is possible that prior DEI training is a mediating factor for differences noted in gender. Future research should consider historical, societal, or environmental factors that may explain if DEI training is more effective among women, or if women are more likely to pursue DEI training. Overall, there was a low correlation between Cultural Awareness and Sensitivity and Culturally Competent Behaviour scores and independent factors (accounting for 15% and 19% variance in outcome, respectively), suggesting there may be numerous yet unidentified variables that contribute to overall cultural competence. Understanding cultural competency may serve to improve clinical interactions and patient outcomes. Specifically, more cross-cultural research is needed to understand patient expectations regarding their clinical experience, which may serve to improve patient outcomes [34]. More research is needed to identify the variables that relate to cultural competency and further explore gender or sex differences and their association with DEI training.

The majority of chiropractors were aware of cultural health disparities in the Canadian health care system. Furthermore, we found a weak negative association between lack of knowledge in observed cultural health disparities and provider knowledge and behaviour. Additionally, we noted significant variability among responses associated with barriers to the provision of chiropractic services. This variability makes it difficult to understand barriers within this study population and identifies an opportunity for future research to focus on region or practice specific needs. Future research should aim to develop and implement specific educational and training resources (e.g., cultural health literacy) for chiropractors.

Unconscious bias refers to discrimination against a group of people that we are not directly aware of that influences judgement [35]. Unconscious biases have been associated with health disparities (e.g., unequal access to care) [36]. Uncertainties expressed for the statement “I think some people have an agenda to look for discrimination even where it does not exist,” points to potential unconscious biases that may influence provision of care [37]. Disagreement with this statement was associated with higher Cultural Awareness and Sensitivity scores, whereas agreement was associated with lower scores. These findings suggest the need for practitioners to identify and understand their unconscious bias, which may be important in facilitating behavioural change. However, it is important to note that surveys are limited in interpreting why uncertainty among respondents exists, which may be due to the difficulty in capturing the complexity of unconscious bias or confusion regarding the question. Future studies should incorporate a mixed methods research design to explore the complexity of cultural competency in more detail.

### Limitations

Recently, the CCA launched a campaign to address cultural competency among Canadian chiropractors, and offered training in diversity, equity, and inclusion where 36% of our sample attended this, which may have led to response bias and potentially overestimating the survey scores. The authors of the CCAI questionnaire recommend the use of the Marlowe-Crowne social desirability scale to account for social desirability, but this was removed to reduce participant burden [22, 23]. Additionally, there were some sections of the survey that were not previously tested, limiting reliability and validity.

## Conclusion

This study provides the first description of cultural competency within the chiropractic profession in Canada. We noted high cultural awareness and sensitivity scores, and lower culturally competent behaviour scores. There were several factors (e.g., gender, race, prior DEI training, etc.) that were weakly associated with cultural competency. Results suggest a gap between knowledge and behaviour and uncover several barriers and challenges that may inform the development of profession-specific training in cultural competence. These findings emphasize the complexity of cultural competence as a social construct.

## Supplementary Information

**Additional file 1: Appendix A.**  Social Equity in Health Care Survey.

**Additional file 2: Appendix B.** Rules for Removal of Duplicates.

## Data Availability

The datasets used and/or analysed during the current study are available from the corresponding author on reasonable request.

## Abbreviations

CCA: Canadian Chiropractic Association
DEI: Diversity, equity, and inclusion
CPA: Canadian Physiotherapy Association
CCAI: Cultural competence assessment instrument
CI: Confidence interval
SD: Standard deviation
