# Supplementary material for: Assessing cultural competency among Canadian chiropractors: a cross-sectional survey of Canadian Chiropractic Association members
Source: Chiropr Man Therap. 2023 Jan 12;31:1. doi: 10.1186/s12998-023-00474-4 (PMC9835226; doi:10.1186/s12998-023-00474-4)
Supplement: Supplementary file 1 — Additional file 1: Appendix A. Social Equity in Health Care Survey. [file 12998_2023_474_MOESM1_ESM.docx]

**Social Equity in Health Care Survey**

**Section 1:**

First, we would like to know more about you.

1. What is your age in years? ____
2. What is your sex assigned at birth?

- Male
- Female
- Prefer not to answer

1. Which gender identity do you most identify with? *Select all that apply.*

- Woman
- Man
- Trans woman
- Trans man
- Gender-fluid or non-binary
- Indigenous or other cultural gender minority identity (e.g. two-spirit)
- An identity not listed (please specify): _______________
- Prefer not to answer

1. What is your sexual orientation?

- Asexual
- Bisexual
- Gay
- Straight (heterosexual)
- Lesbian
- Pansexual
- Queer
- Questioning or unsure
- Same-gender loving
- An identity not listed (please specify): ______________
- Prefer not to answer

1. Do you identify as an Indigenous person, that is, First Nations (status or non-status), Métis or Inuk (Inuit) or as having Indigenous ancestry?

- Yes
- No
- Do not know
- Prefer not to answer

1. What race do you identify yourself with? *Select all that apply.*

- Arab
- Black
- White/Caucasian
- Chinese
- Filipino
- Japanese
- Korean
- Latin American
- South Asian (e.g. East Indian, Pakistani, Sri Lankan, etc.)
- Southeast Asian (e.g. Vietnamese, Cambondian, Thai, etc.)
- West Asian (e.g. Iranian, Afghan, etc.)
- Mixed race
- Another visible minority group (please specify): ______________
- Prefer not to answer

1. Please indicate your ethnicity. *Select all that apply.*

- African – Central or West (e.g. Ghanaian, Liberian, Nigerian, Senegalese)
- African – Northern (e.g. Egyptian, Libyan, Tunisian)
- African – Southern or Eastern (e.g. Ethiopian, Kenyan, South African, Ugandan)
- American
- Asian – West, Central or Middle Eastern (e.g. Afghan, Iranian, Iraqi, Israeli, Lebanese)
- Asian – South (e.g. Bengali, Punjabi, Sri Lankan, Tamil)
- Asian – East or Southeast (e.g. Chinese, Filipino, Japanese, Korean, Vietnamese)
- Canadian
- Caribbean (e.g. Cuban, Dominican, Jamaican, West Indian)
- European – British Isles (e.g. English, Irish, Scottish)
- European – French (e.g. Breton, French)
- European – Western (e.g. Austrian, Dutch, German)
- European – Northern (e.g. Danish, Swedish, Norwegian)
- European – Eastern (e.g. Czech, Hungarian, Polish, Ukrainian)
- European – Southern (e.g. Croatian, Greek, Italian, Portuguese, Spanish)
- Indigenous (First Nations, Inuit, Métis)
- Latin, Central and South American (e.g. Argentinian, Brazilian, Mexican)
- Oceana (Australian and New Zealand)
- Pacific Islands (e.g. Fijian, Hawaiian, Samoan)
- Mixed ethnicity
- Another (please specify): _____

1. Are you a person with a disability?

*The United Nations Convention on the Rights of Persons with Disabilities defines persons with disabilities as “those who have long-term physical, mental, intellectual or sensory impairments which in interaction with various barriers may hinder their full and effective participation in society on an equal basis with others”. This may include (but is not limited to) persistent or episodic physical, cognitive, psychosocial/mental health, sensory, developmental/intellectual or learning impairments; and difficulty hearing, seeing, communicating, walking, climbing stairs, bending, learning or doing any similar daily activity.*

- Yes
- No
- Prefer not to answer

If yes, what type of disability do you have? *Check all that apply.*

- Cognitive (i.e. learning, developmental, memory)
- Mental health-related
- Physical (i.e. mobility, flexibility, dexterity, pain)
- Sensory (i.e. seeing, hearing)
- Another (please list):

1. Are you proficient in another language aside from English?

- Yes
- No (skip to question 12)

1. If you are proficient in another language, which language(s) do you speak? Below is a list of the top ten languages spoken in Canada (aside from English) based on Census data.

- French
- Canadian Indigenous languages
- Punjabi
- Farsi
- Spanish
- Italian
- German
- Cantonese
- Tagalog
- Arabic
- Mandarin
- Portuguese
- Another (please specify): ______________

1. If you are proficient in another language, do you use this language to communicate with patients (e.g. assessments, follow-up, etc.)?

- Yes
- No

**Section 2**

In this section, we ask you to provide information related to your practice.

1. How many years of clinical experience do you have? ____
2. In which province/territory do you primarily practice?

- Alberta
- British Columbia
- Manitoba
- New Brunswick
- Newfoundland and Labrador
- Northwest Territories
- Nova Scotia
- Nunavut
- Ontario
- Prince Edward Island
- Quebec
- Saskatchewan
- Yukon

1. What provincial college are you registered with?_____
2. Which of the following best describes the community where you primarily practice?

- Rural/remote region (population: ~1000 to 10,000)
- Town or smaller regional city (population: ~10,000 to 100,000)
- Major city (urban/metropolitan/suburban) (population: >100,000)

1. What kind of clinical setting do you practice in (check all that apply)?

- Community clinic – Solo practitioner (chiropractor/occupational therapists/physiotherapist only)
- Community clinic – Interdisciplinary rehabilitation clinic (i.e. chiropractic/occupational therapy/physiotherapy offered alongside other rehabilitation disciplines such as or chiropractic, physiotherapy or occupational therapy)
- Community clinic – Interdisciplinary medical practice (i.e. chiropractic/ occupational therapy/physiotherapy offered alongside physicians and/or specialists)
- Community clinic – Interdisciplinary Complementary and Alternative Medicine clinic (i.e. chiropractic/occupational therapy/physiotherapy offered alongside other complementary and alternative therapies such as osteopathy, naturopathy, homeopathy and/or massage therapy)
- Hospital outpatient
- Hospital inpatient
- Other (specify): ___________

**Section 3**

In this section, we ask you to think about your personal feelings toward a series of statements.

For each of the following statements, please select the response that best describes how you feel about the statement.

1. Race is the most important factor in determining a person’s culture.

| Strongly  Agree | Agree | Somewhat  Agree | Neutral | Somewhat  Disagree | Disagree | Strongly  Disagree | No Opinion |
| --- | --- | --- | --- | --- | --- | --- | --- |
| ❑ | ❑ | ❑ | ❑ | ❑ | ❑ | ❑ | ❑ |

1. People with a common cultural background think and act alike.

| Strongly  Agree | Agree | Somewhat  Agree | Neutral | Somewhat  Disagree | Disagree | Strongly  Disagree | No Opinion |
| --- | --- | --- | --- | --- | --- | --- | --- |
| ❑ | ❑ | ❑ | ❑ | ❑ | ❑ | ❑ | ❑ |

1. Many aspects of culture influence health and health care.

| Strongly  Agree | Agree | Somewhat  Agree | Neutral | Somewhat  Disagree | Disagree | Strongly  Disagree | No Opinion |
| --- | --- | --- | --- | --- | --- | --- | --- |
| ❑ | ❑ | ❑ | ❑ | ❑ | ❑ | ❑ | ❑ |

1. Aspects of cultural diversity need to be assessed for each individual, group, and organization.

| Strongly  Agree | Agree | Somewhat  Agree | Neutral | Somewhat  Disagree | Disagree | Strongly  Disagree | No Opinion |
| --- | --- | --- | --- | --- | --- | --- | --- |
| ❑ | ❑ | ❑ | ❑ | ❑ | ❑ | ❑ | ❑ |

1. If I know about a person’s culture, I don’t need to assess their personal preferences for health services.

| Strongly  Agree | Agree | Somewhat  Agree | Neutral | Somewhat  Disagree | Disagree | Strongly  Disagree | No Opinion |
| --- | --- | --- | --- | --- | --- | --- | --- |
| ❑ | ❑ | ❑ | ❑ | ❑ | ❑ | ❑ | ❑ |

1. Spiritual and religious beliefs are important aspects of many cultural groups.

| Strongly  Agree | Agree | Somewhat  Agree | Neutral | Somewhat  Disagree | Disagree | Strongly  Disagree | No Opinion |
| --- | --- | --- | --- | --- | --- | --- | --- |
| ❑ | ❑ | ❑ | ❑ | ❑ | ❑ | ❑ | ❑ |

1. Individual people may identify with more than one cultural group.

| Strongly  Agree | Agree | Somewhat  Agree | Neutral | Somewhat  Disagree | Disagree | Strongly  Disagree | No Opinion |
| --- | --- | --- | --- | --- | --- | --- | --- |
| ❑ | ❑ | ❑ | ❑ | ❑ | ❑ | ❑ | ❑ |

1. Language barriers are the only difficulties for recent immigrants to Canada.

| Strongly  Agree | Agree | Somewhat  Agree | Neutral | Somewhat  Disagree | Disagree | Strongly  Disagree | No Opinion |
| --- | --- | --- | --- | --- | --- | --- | --- |
| ❑ | ❑ | ❑ | ❑ | ❑ | ❑ | ❑ | ❑ |

1. I believe that everyone should be treated with respect no matter what their cultural heritage.

| Strongly  Agree | Agree | Somewhat  Agree | Neutral | Somewhat  Disagree | Disagree | Strongly  Disagree | No Opinion |
| --- | --- | --- | --- | --- | --- | --- | --- |
| ❑ | ❑ | ❑ | ❑ | ❑ | ❑ | ❑ | ❑ |

1. I understand that people from different cultures may define the concept of “health care” in different ways.

| Strongly  Agree | Agree | Somewhat  Agree | Neutral | Somewhat  Disagree | Disagree | Strongly  Disagree | No Opinion |
| --- | --- | --- | --- | --- | --- | --- | --- |
| ❑ | ❑ | ❑ | ❑ | ❑ | ❑ | ❑ | ❑ |

1. I think that knowing about different cultural groups helps direct my work with individuals, families, groups, and organizations.

| Strongly  Agree | Agree | Somewhat  Agree | Neutral | Somewhat  Disagree | Disagree | Strongly  Disagree | No Opinion |
| --- | --- | --- | --- | --- | --- | --- | --- |
| ❑ | ❑ | ❑ | ❑ | ❑ | ❑ | ❑ | ❑ |

**Section 4**

For the following questions, we would like you to think about your day to day activities within your practice and tell us how often you perform a number of tasks.

For each of the following statements, please select the response that best describes how often you do the following:

1. I include cultural assessment when I do patient evaluations.

| Always | Very Often | Often | Somewhat  Often | Sometimes | Few Times | Never | Not sure |
| --- | --- | --- | --- | --- | --- | --- | --- |
| ❑ | ❑ | ❑ | ❑ | ❑ | ❑ | ❑ | ❑ |

1. I seek information on cultural needs when I identify new people in my practice.

| Always | Very Often | Often | Somewhat  Often | Sometimes | Few Times | Never | Not sure |
| --- | --- | --- | --- | --- | --- | --- | --- |
| ❑ | ❑ | ❑ | ❑ | ❑ | ❑ | ❑ | ❑ |

1. I have resource books and other materials available to help me learn about people from different cultures.

| Always | Very Often | Often | Somewhat  Often | Sometimes | Few Times | Never | Not sure |
| --- | --- | --- | --- | --- | --- | --- | --- |
| ❑ | ❑ | ❑ | ❑ | ❑ | ❑ | ❑ | ❑ |

1. I use a variety of sources to learn about the cultural heritage of others.

| Always | Very Often | Often | Somewhat  Often | Sometimes | Few Times | Never | Not sure |
| --- | --- | --- | --- | --- | --- | --- | --- |
| ❑ | ❑ | ❑ | ❑ | ❑ | ❑ | ❑ | ❑ |

1. I ask patients to tell me about their own explanations of health and illness.

| Always | Very Often | Often | Somewhat  Often | Sometimes | Few Times | Never | Not sure |
| --- | --- | --- | --- | --- | --- | --- | --- |
| ❑ | ❑ | ❑ | ❑ | ❑ | ❑ | ❑ | ❑ |

1. I ask patients to tell me about their expectations for health services.

| Always | Very Often | Often | Somewhat  Often | Sometimes | Few Times | Never | Not sure |
| --- | --- | --- | --- | --- | --- | --- | --- |
| ❑ | ❑ | ❑ | ❑ | ❑ | ❑ | ❑ | ❑ |

1. I avoid using generalizations to stereotype groups of patients.

| Always | Very Often | Often | Somewhat  Often | Sometimes | Few Times | Never | Not sure |
| --- | --- | --- | --- | --- | --- | --- | --- |
| ❑ | ❑ | ❑ | ❑ | ❑ | ❑ | ❑ | ❑ |

1. I recognize potential barriers to service that might be encountered by different patients from diverse groups.

| Always | Very Often | Often | Somewhat  Often | Sometimes | Few Times | Never | Not sure |
| --- | --- | --- | --- | --- | --- | --- | --- |
| ❑ | ❑ | ❑ | ❑ | ❑ | ❑ | ❑ | ❑ |

1. I remove obstacles for patients of different cultures when I identify barriers to services.

| Always | Very Often | Often | Somewhat  Often | Sometimes | Few Times | Never | Not sure |
| --- | --- | --- | --- | --- | --- | --- | --- |
| ❑ | ❑ | ❑ | ❑ | ❑ | ❑ | ❑ | ❑ |

1. I remove obstacles for patients of different cultures when people identify barriers to me.

| Always | Very Often | Often | Somewhat  Often | Sometimes | Few Times | Never | Not sure |
| --- | --- | --- | --- | --- | --- | --- | --- |
| ❑ | ❑ | ❑ | ❑ | ❑ | ❑ | ❑ | ❑ |

1. I welcome feedback from patients about how I relate to people from different cultures.

| Always | Very Often | Often | Somewhat  Often | Sometimes | Few Times | Never | Not sure |
| --- | --- | --- | --- | --- | --- | --- | --- |
| ❑ | ❑ | ❑ | ❑ | ❑ | ❑ | ❑ | ❑ |

1. I find ways to adapt my services to individual and group cultural preferences.

| Always | Very Often | Often | Somewhat  Often | Sometimes | Few Times | Never | Not sure |
| --- | --- | --- | --- | --- | --- | --- | --- |
| ❑ | ❑ | ❑ | ❑ | ❑ | ❑ | ❑ | ❑ |

1. I document cultural assessments if I provide direct patient services.

| Always | Very Often | Often | Somewhat  Often | Sometimes | Few Times | Never | Not sure |
| --- | --- | --- | --- | --- | --- | --- | --- |
| ❑ | ❑ | ❑ | ❑ | ❑ | ❑ | ❑ | ❑ |

1. I document the adaptations I make with patients if I provide direct patient services.

| Always | Very Often | Often | Somewhat  Often | Sometimes | Few Times | Never | Not sure |
| --- | --- | --- | --- | --- | --- | --- | --- |
| ❑ | ❑ | ❑ | ❑ | ❑ | ❑ | ❑ | ❑ |

**Section 5**

In the following questions, we will ask you to think about how often you have observed cultural health disparities. *A cultural health disparity is a difference in health that is closely linked with disadvantages experienced by a person based on their belonging to a particular community or cultural group.*

For each of the following statements, please select the response that best describes how often you have observed the following.

1. I have observed cultural health disparities in pain outcomes.

| Always | Very Often | Often | Somewhat  Often | Sometimes | Few Times | Never | Not sure |
| --- | --- | --- | --- | --- | --- | --- | --- |
| ❑ | ❑ | ❑ | ❑ | ❑ | ❑ | ❑ | ❑ |

1. I have observed cultural health disparities in clinical outcomes.

| Always | Very Often | Often | Somewhat  Often | Sometimes | Few Times | Never | Not sure |
| --- | --- | --- | --- | --- | --- | --- | --- |
| ❑ | ❑ | ❑ | ❑ | ❑ | ❑ | ❑ | ❑ |

1. I have observed cultural health disparities in satisfaction with care.

| Always | Very Often | Often | Somewhat  Often | Sometimes | Few Times | Never | Not sure |
| --- | --- | --- | --- | --- | --- | --- | --- |
| ❑ | ❑ | ❑ | ❑ | ❑ | ❑ | ❑ | ❑ |

1. I have observed cultural health disparities in overall health status due to patients’ cultural differences.

| Always | Very Often | Often | Somewhat  Often | Sometimes | Few Times | Never | Not sure |
| --- | --- | --- | --- | --- | --- | --- | --- |
| ❑ | ❑ | ❑ | ❑ | ❑ | ❑ | ❑ | ❑ |

**Section 6**

In this section, we are asking you to think about some of the challenges you may or may not have experienced in trying to deliver care to patients from diverse groups.

For each of the following statements please select the response that best describes how you feel about the statement.

1. The *cost of my services* limits me from providing care for people from some communities and/or cultural groups.

|  |  |  |  |  |  |  |  |
| --- | --- | --- | --- | --- | --- | --- | --- |
|  |  |  |  |  |  |  |  |
| Strongly  Agree | Agree | Somewhat  Agree | Neutral | Somewhat  Disagree | Disagree | Strongly  Disagree | No Opinion |
| ❑ | ❑ | ❑ | ❑ | ❑ | ❑ | ❑ | ❑ |

1. I find it difficult to develop treatment plans that meet the diverse needs of the people in my community.

|  |  |  |  |  |  |  |  |
| --- | --- | --- | --- | --- | --- | --- | --- |
|  |  |  |  |  |  |  |  |
| Strongly  Agree | Agree | Somewhat  Agree | Neutral | Somewhat  Disagree | Disagree | Strongly  Disagree | No Opinion |
| ❑ | ❑ | ❑ | ❑ | ❑ | ❑ | ❑ | ❑ |

1. Patients from some communities and/or cultural groups have difficulty complying with my treatment recommendations (e.g., number of treatment sessions, adherence to exercise, etc.).

|  |  |  |  |  |  |  |  |
| --- | --- | --- | --- | --- | --- | --- | --- |
|  |  |  |  |  |  |  |  |
| Strongly  Agree | Agree | Somewhat  Agree | Neutral | Somewhat  Disagree | Disagree | Strongly  Disagree | No Opinion |
| ❑ | ❑ | ❑ | ❑ | ❑ | ❑ | ❑ | ❑ |

1. I find it difficult to build rapport with patients who are different from me.

|  |  |  |  |  |  |  |  |
| --- | --- | --- | --- | --- | --- | --- | --- |
|  |  |  |  |  |  |  |  |
| Strongly  Agree | Agree | Somewhat  Agree | Neutral | Somewhat  Disagree | Disagree | Strongly  Disagree | No Opinion |
| ❑ | ❑ | ❑ | ❑ | ❑ | ❑ | ❑ | ❑ |

1. Patients from some communities and/or cultural groups have difficulty trusting the health care system.

|  |  |  |  |  |  |  |  |
| --- | --- | --- | --- | --- | --- | --- | --- |
|  |  |  |  |  |  |  |  |
| Strongly  Agree | Agree | Somewhat  Agree | Neutral | Somewhat  Disagree | Disagree | Strongly  Disagree | No Opinion |
| ❑ | ❑ | ❑ | ❑ | ❑ | ❑ | ❑ | ❑ |

1. Language barriers prevent me from providing an informed consent and educating some of my patients to the extent that I would like.

|  |  |  |  |  |  |  |  |
| --- | --- | --- | --- | --- | --- | --- | --- |
|  |  |  |  |  |  |  |  |
| Strongly  Agree | Agree | Somewhat  Agree | Neutral | Somewhat  Disagree | Disagree | Strongly  Disagree | No Opinion |
| ❑ | ❑ | ❑ | ❑ | ❑ | ❑ | ❑ | ❑ |

1. Language barriers impact some of my patients’ ability to comply with care instructions.

|  |  |  |  |  |  |  |  |
| --- | --- | --- | --- | --- | --- | --- | --- |
|  |  |  |  |  |  |  |  |
| Strongly  Agree | Agree | Somewhat  Agree | Neutral | Somewhat  Disagree | Disagree | Strongly  Disagree | No Opinion |
| ❑ | ❑ | ❑ | ❑ | ❑ | ❑ | ❑ | ❑ |

**Section 7**

The following questions pertain to previous training you may have received in cultural competence/diversity. You will also be asked about how you manage issues related to diversity, equity and inclusion in your practice and in the community where you live or work.

1. Have you ever participated in cultural diversity training?

- Yes
- No (If no, skip to question 54)

1. If you have had prior diversity training, which option(s) below best describe(s) it?

*(Check all that apply)*

- Specific college/university credit course related to diversity, equity and inclusion
- Content covered in a college/university course
- Professional Conference or Seminar
- Employer Sponsored Program
- Continuing Education Offering
- CCA (Canadian Chiropractic Association)/CPA (Canadian Physiotherapy Association)/COTA (Canadian Occupational Therapy Association) Diversity, Equity, and Inclusion Training
- Other diversity training types (Specify) _____________________________

1. Were any of the following concepts addressed during your chiropractic/occupational therapy/physiotherapy education? (Check all that apply)

- Racial and/or ethnic predispositions to genetic-specific illness and disorders.
- Biological differences between males and females in sex-specific illness and disorders.
- Psychological and social stresses of living in a discriminatory society
- Cultural health disparities and utilization of health care services
- Cultural health disparities in socioeconomic status, health status and language barriers
- Cultural health disparities in access to social services, education, employment, and health services.
- Potential for bias in health care delivery to people from diverse communities and/or cultural groups.
- Institutionalized racial and ethnic biases
- Institutionalized sex and gender roles
- Intersectionality of the social determinants of health (e.g., race, gender, sexual orientation, ethnicity, etc.)
- None

1. Please select the response that best describes how you feel about the following statements.
2. I am uncertain about how to approach cultural competence with my staff/colleagues.

| Strongly  Agree | Agree | Somewhat  Agree | Neutral | Somewhat  Disagree | Disagree | Strongly  Disagree | No Opinion |
| --- | --- | --- | --- | --- | --- | --- | --- |
| ❑ | ❑ | ❑ | ❑ | ❑ | ❑ | ❑ | ❑ |

1. I am uncertain about how to provide culturally sensitive care to my patients.

| Strongly  Agree | Agree | Somewhat  Agree | Neutral | Somewhat  Disagree | Disagree | Strongly  Disagree | No Opinion |
| --- | --- | --- | --- | --- | --- | --- | --- |
| ❑ | ❑ | ❑ | ❑ | ❑ | ❑ | ❑ | ❑ |

1. I am uncertain whether my actions or words will be seen as not meeting current standards in diversity, equity and inclusion.

| Strongly  Agree | Agree | Somewhat  Agree | Neutral | Somewhat  Disagree | Disagree | Strongly  Disagree | No Opinion |
| --- | --- | --- | --- | --- | --- | --- | --- |
| ❑ | ❑ | ❑ | ❑ | ❑ | ❑ | ❑ | ❑ |

1. I think some people have an agenda to look for discrimination even where it does not exist.

| Strongly  Agree | Agree | Somewhat  Agree | Neutral | Somewhat  Disagree | Disagree | Strongly  Disagree | No Opinion |
| --- | --- | --- | --- | --- | --- | --- | --- |
| ❑ | ❑ | ❑ | ❑ | ❑ | ❑ | ❑ | ❑ |

1. I do my best to hire staff, or advocate for the hiring of staff, that are representative of the community in which I practice.

| Strongly  Agree | Agree | Somewhat  Agree | Neutral | Somewhat  Disagree | Disagree | Strongly  Disagree | No Opinion |
| --- | --- | --- | --- | --- | --- | --- | --- |
| ❑ | ❑ | ❑ | ❑ | ❑ | ❑ | ❑ | ❑ |

1. I accommodate patients who speak a language other than my own (e.g. invite a family member or staff member to translate, translate intake and educational materials, use of translation apps/software, etc.)

| Strongly  Agree | Agree | Somewhat  Agree | Neutral | Somewhat  Disagree | Disagree | Strongly  Disagree | No Opinion |
| --- | --- | --- | --- | --- | --- | --- | --- |
| ❑ | ❑ | ❑ | ❑ | ❑ | ❑ | ❑ | ❑ |

1. I promote equity and inclusion among staff and colleagues (e.g. arranging or participating in cultural competency training, etc.)

| Strongly  Agree | Agree | Somewhat  Agree | Neutral | Somewhat  Disagree | Disagree | Strongly  Disagree | No Opinion |
| --- | --- | --- | --- | --- | --- | --- | --- |
| ❑ | ❑ | ❑ | ❑ | ❑ | ❑ | ❑ | ❑ |

1. How likely are you to engage in activities to improve your skills and competence in providing inclusive and equitable chiropractic/physiotherapy/occupational therapy care to patients?

| Very likely | Moderately likely | Neither likely nor unlikely | Moderately unlikely | Very Unlikely | No Opinion |
| --- | --- | --- | --- | --- | --- |
| ❑ | ❑ | ❑ | ❑ | ❑ | ❑ |

1. Considering next steps, what are the top 3 suggestions that you have for your professional association to help you incorporate cultural competence and agility in your practice? ________________________________________________________________________________________________________________________________________________________________________________________________________________________
2. As part of our future research, we will be conducting semi-structured qualitative interviews to better understand clinicians’ perspectives of diversity, equity and inclusion within the profession. If you are interested in being a part of this study and would like more information please leave your contact information or email Dr. Nora Bakaa at bakaanh@mcmaster.ca

Email:

1. To thank you for participating in our survey, we would like to provide you with the opportunity to win one of ten vouchers to cover one year of your CCA membership dues/or one of five $50 gift certificates. If you are interested in being entered into the draw, please click on the following link: *<*Insert Survey Link*>

*Draw survey link:

To thank you for participating in our survey, we would like to provide you with the opportunity to win one of ten vouchers to cover one year of your CCA membership dues/or one of five $50 gift certificates. If you are interested in being entered into the draw, please enter your email address: __________
