# Supplementary material for: Assessing cultural competency among Canadian chiropractors: a cross-sectional survey of Canadian Chiropractic Association members
Source: Chiropr Man Therap. 2023 Jan 12;31:1. doi: 10.1186/s12998-023-00474-4 (PMC9835226; doi:10.1186/s12998-023-00474-4)
Supplement: Supplementary file 2 — Additional file 2: Appendix B. Rules for Removal of Duplicates. [file 12998_2023_474_MOESM2_ESM.docx]

**Appendix B: Removal of Duplicates**

Rules:

1. If response ID’s identical, all data is identical and one response will be deleted.
2. If no data was collected (i.e. respondent did not progress past page 0), the duplicate without data will be deleted.
3. If the demographics are identical, incomplete responses will be deleted.
4. If demographics are identical and multiple complete responses were submitted, only the final response will be retained.
5. Remaining duplicate IP’s with unique demographics will be considered unique responses.

*Incomplete responses removed with Rule 3:

Survey Page 1 - 21

Survey Page 2 - 35

Survey Page 3 - 7

Survey Page 4 - 10

Survey Page 5 - 1

Survey Page 6 - 17
